# Changes in Caregiver Burden Following Unilateral Magnetic Resonance‐Guided Focused Ultrasound Thalamotomy for Essential Tremor

**DOI:** 10.1002/mdc3.14034

**Published:** 2024-04-04

**Authors:** Georgia Gopinath, Nadia Scantlebury, Isabella J. Sewell, Camryn R. Rohringer, Shayan Sivadas, Melissa McSweeney, Silina Z. Boshmaf, Benjamin Lam, Clement Hamani, Agessandro Abrahao, Michael L. Schwartz, Nir Lipsman, Jennifer S. Rabin

**Affiliations:** ^1^ Hurvitz Brain Sciences Program Sunnybrook Research Institute Toronto ON Canada; ^2^ Harquail Centre for Neuromodulation Sunnybrook Research Institute Toronto ON Canada; ^3^ Division of Neurology, Department of Medicine, Sunnybrook Health Sciences Centre University of Toronto Toronto ON Canada; ^4^ Division of Neurosurgery, Department of Surgery, Sunnybrook Health Sciences Centre University of Toronto Toronto ON Canada; ^5^ Department of Psychiatry, Sunnybrook Health Sciences Centre University of Toronto Toronto ON Canada; ^6^ Rehabilitation Sciences Institute, University of Toronto Toronto ON Canada

**Keywords:** essential tremor, thalamotomy, focused ultrasound, caregiver burden

Essential tremor (ET) is characterized by bilateral action tremor that predominantly affects the upper limbs. Severe tremor can influence patients’ ability to perform activities of daily living (ADLs), often requiring reliable caregiver support. Family members commonly take on the role of informal caregivers, with the burden increasing with more caregiving tasks.^1^

Up to 50% of patients with ET do not respond well to first‐line medical therapies.^2^ In such cases, surgical options such as magnetic resonance‐guided focused ultrasound (MRgFUS) thalamotomy can be considered. MRgFUS thalamotomy reduces upper limb tremor and improves ADL performance and psychosocial functioning.^3^, ^4^ However, it remains unclear whether MRgFUS also affects the load and burden experienced by caregivers. Here, we examined whether MRgFUS thalamotomy for ET influences caregiver assistance with ADLs and overall caregiver burden.

We included a sample of convenience of 18 individuals with medication‐refractory ET scheduled for unilateral MRgFUS thalamotomy [mean age = 71 ± 8 years, female = 7 (39%)] and their caregivers [mean age = 66 ± 13 years, female = 12 (67%)]. Participants identified their primary caregiver, which was defined as the individual providing the most assistance with tremor‐related challenges. Fourteen of the caregivers were spouses, one was a partner living separately from the patient, and three were children of the patients (Table S1). We assessed tremor severity of the treated hand using Parts A and B of the Clinical Rating Scale for Tremor (CRST), after excluding the handwriting item. ADL assistance was quantified as the number of tasks caregivers assisted with from a list of 10 tasks (Fig. 1A and S1).^1^ Global caregiver burden was measured using the 12‐item Zarit Burden Interview Short Form (ZBI‐12), which asks about emotional, physical, and social strains experienced by caregivers (Fig. 1B and S2).^5^ ET‐specific caregiver burden was assessed with a five‐item questionnaire assessing concerns associated with caregiving for individuals with ET (Fig. 1C and S3).^1^ For the ZBI‐12 and ET‐specific caregiver scale, higher scores represent greater burden. Assessments were completed at baseline and approximately 4 months following MRgFUS (Table S1). Changes from baseline were tested using paired‐sample *t*‐tests or *Wilcoxon* tests, and associations were assessed using Pearson or Spearman correlations, as appropriate.

**Figure 1:**
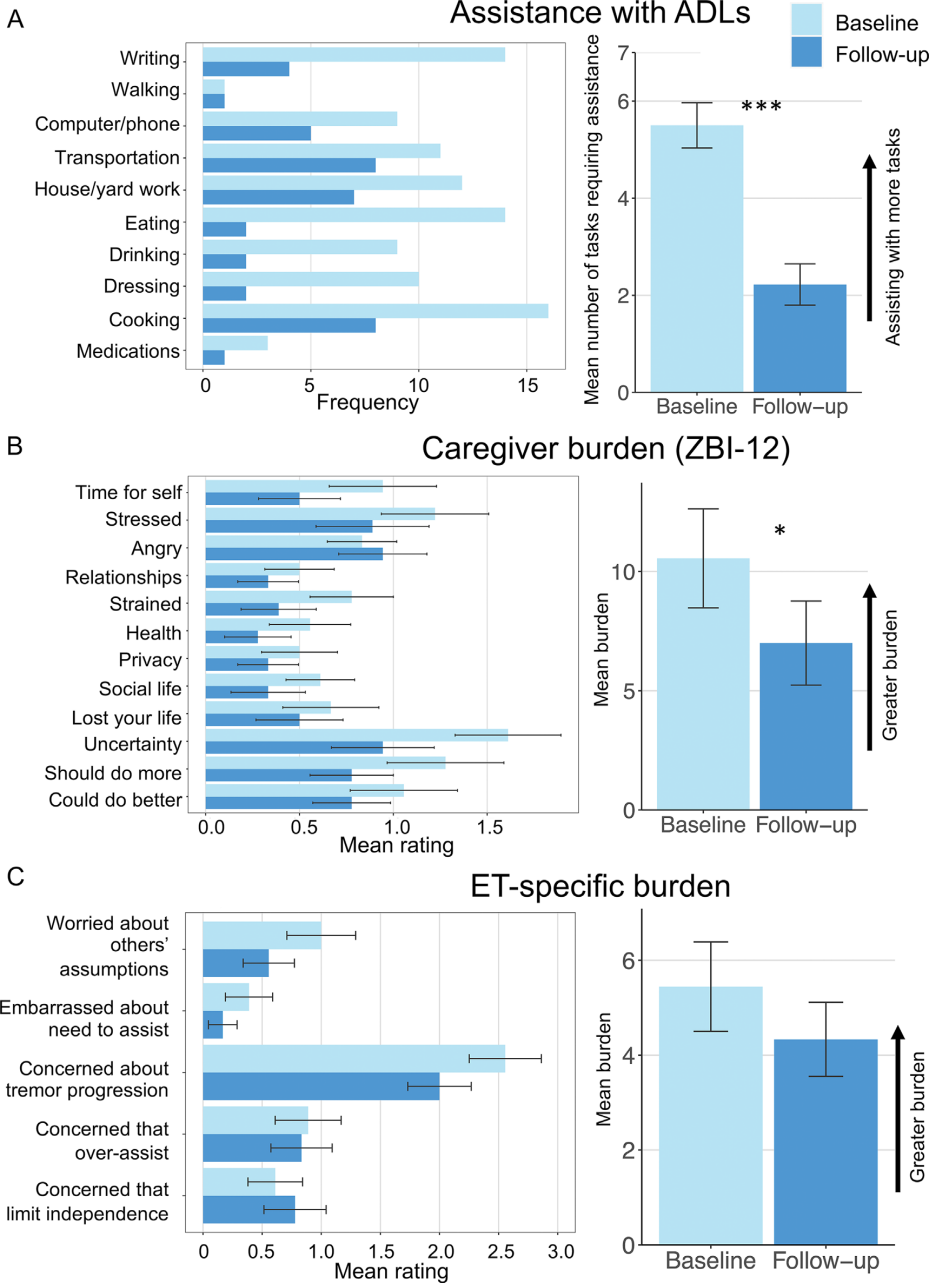
Effects of MRgFUS thalamotomy on caregiver assistance with activities of daily living (ADLs) and caregiver burden. (**A**) The left plot displays the number of caregivers who reported assisting with each ADL at baseline and follow‐up; the right plot illustrates the mean number of ADLs caregivers assisted with at baseline and follow‐up. (**B**) The left plot depicts the mean rating for each item of the ZBI‐12 at baseline and follow‐up; the right plot displays the mean total ZBI‐12 score at baseline and follow‐up. (**C**) The left plot shows the mean rating for each item on the ET‐specific caregiver burden scale at baseline and follow‐up; the right plot depicts the mean total score on the ET‐specific caregiver burden scale at baseline and follow‐up. Error bars represent standard error. *P < 0.05, ***P < 0.001.

At baseline, the mean tremor score on the CRST was 17.8 ± 4.5. Following MRgFUS, there was a significant reduction in tremor severity (*M* = 8.7 ± 6.5, *t* = 7.6, Hedges’ g = 1.7, *P* < 0.001), and an average tremor improvement of 54% ± 31%. At baseline, caregivers assisted with a mean of 5.5 ± 2.0 ADLs. The most common tasks requiring assistance at baseline included cooking (89% of caregivers reported assisting), eating (78%), writing (78%), and house/yard work (67%). Following MRgFUS, caregivers assisted with significantly fewer activities (*M* = 2.2 ± 1.8 ADLs, *V* = 149, *r* = 1.43, *P* < 0.001; Fig. 1A), including cooking (44%), eating (11%), writing (22%), and house/yard work (39%). Of note, percent reduction in tremor severity and reduction in ADL assistance were significantly associated (rho = 0.48, *P* = 0.05, Fig. S4).

The mean ZBI‐12 score at baseline was 10.6 ± 8.8, substantially exceeding previous reports for individuals with ET (3.0–6.4).^6^, ^7^ This is expected given that MRgFUS thalamotomy is typically considered for individuals with severe and refractory tremor.^8^ Following MRgFUS, caregivers endorsed significantly less burden on the ZBI‐12 (*M* = 7.0 ± 7.5, *t* = 2.7, Hedges’ g = 0.6, *P* = 0.015; Fig. 1B). Reduction in caregiver burden on the ZBI‐12 was not associated with reduction in tremor severity (*r* = 0.04, *P* = 0.88, Fig. S4).

Relative to baseline (*M* = 5.4 ± 4.0), there was no significant post‐operative change in ET‐specific caregiver burden (*M* = 4.3 ± 3.3, *t* = 1.2, Hedges’ g = 0.3, *P* = 0.26; Fig. 1C). This could be due to the questionnaire's focus on complex concerns associated with ET caregiving (eg, over‐assisting, limiting patient's independence), which may not change following MRgFUS. Reduction in tremor severity and change in ET‐specific burden were not significantly associated (*r* = 0.18, *P* = 0.47, Fig. S4). Both before and after MRgFUS, the most frequently endorsed questionnaire item was: “*Do you feel concerned about how your relative's tremor will progress over time?*” (endorsed by 15/18 caregivers at baseline and 13/18 caregivers post‐operatively), indicating a persistent concern about the potential worsening of tremor.

To summarize, MRgFUS thalamotomy not only reduces the need for assistance with daily tasks, but also decreases the burden experienced by caregivers in the months following the procedure. There was a significant association between reduced tremor severity and decreased assistance with ADLs, but not caregiver burden. This finding suggests that tremor severity directly impacts the need for practical support, while its relationship with caregiver burden is more nuanced. It is possible that caregiver burden in ET is more affected by caregivers’ perception of patients’ distress than solely by tremor severity.^1^ Due to the limited sample size, we were unable to investigate the influence of poor tremor outcomes and side effects on caregiver burden. Additionally, the follow‐up period was relatively short (ie, 4 months). Thus, some patients might have experienced side effects that could resolve over time and improve caregiver burden. Future studies with larger samples sizes and longer follow‐up durations are needed. In conclusion, the benefits of MRgFUS thalamotomy extend beyond the patients to positively impact their caregivers’ experiences.

## Author Roles

(1) Research Project: A. Conception, B. Organization, C. Execution; (2) Statistical Analysis: A. Design, B. Execution, C. Review and Critique; (3) Manuscript Preparation: A. Writing of the First Draft, B. Review and Critique.

G.G.: 1A, 1B, 1C, 2A, 2B, 2C, 3A

N.S.: 1A, 1B, 1C, 2A, 2B, 2C, 3A

I.J.S.: 1B, 1C, 2C, 3B

C.R.R.: 1B, 1C, 2C, 3B

S.S.: 1B, 1C, 2C, 3A, 3B

M.M.: 1A, 1B, 1C, 3B

S.Z.B.: 1B, 1C, 3B

B.L.: 1C, 3B

C.H.: 1C, 3B

A.A.: 1C, 3B

M.L.S.: 1C, 3B

N.L.: 1C, 3B

J.S.R.: 1A, 1B, 2A, 2B, 2C, 3B

## Disclosures

**Ethical Compliance Statement:** All activities were performed under approval of the Sunnybrook Research Ethics Board (Project ID 2106). Written informed consent was obtained from each patient prior to participation. We confirm that we read the Journal's position on issues involved in ethical publication and affirm that this work is consistent with those guidelines.

**Funding Sources and Conflicts of Interest:** This work was funded by the University of Toronto, Division of Neurology, Slamen‐Fast New Initiatives. The authors declare that there are no conflicts of interest relevant to this work.

**Funding Disclosures for Previous 12 Months:** C.H. receives support from CIHR, New Frontiers in Research Fund, Harquail Centre for Neuromodulation. A.A. receives support from the Multiple Sclerosis Society of Canada, the Focused Ultrasound Foundation, ALS Society of Canada, Weston Brain Institute, and an honorarium from Mitsubishi Tanabe Pharma and Amylyx, Harquail Centre for Neuromodulation. N.L. receives support from CIHR, InSightec, New Frontiers in Research Fund, Veteran's Affairs Canada, Power Corporation, the Alternate Planning Fund, the Multiple Sclerosis Society of Canada, the Focused Ultrasound Foundation, Harquail Centre for Neuromodulation. J.S.R. receives support from CIHR, NSERC, New Frontiers in Research Fund, Alzheimer's Society of Canada, Alzheimer's Association, Harquail Centre for Neuromodulation, Dr. Sandra Black Centre for Brain Resilience & Recovery.

## Supporting information


**Table S1.** Demographics and clinical characteristics.
**Figure S1.** Questions from activities of daily living scale.
**Figure S2.** Questions from Zarit Burden Interview short form.
**Figure S3.** Questions from ET‐specific caregiver burden scale.
**Figure S4.** Correlations between tremor severity and questionnaire outcomes.
