# Supplementary material for: Changes in Caregiver Burden Following Unilateral Magnetic Resonance‐Guided Focused Ultrasound Thalamotomy for Essential Tremor
Source: Mov Disord Clin Pract. 2024 Apr 4;11(7):905–8. doi: 10.1002/mdc3.14034 (PMC11233863; doi:10.1002/mdc3.14034)
Supplement: Supplementary file 1 — Table S1. Demographics and clinical characteristics. Figure S1. Questions from activities of daily living scale. Figure S2. Questions from Zarit Burden Interview short form. Figure S3. Questions from ET‐specific caregiver burden scale. Figure S4. Correlations between tremor severity and questionnaire outcomes. [file MDC3-11-905-s001.docx]

**Supplementary Material**

Table S1: Demographics and Clinical Characteristics…………………………...………………………………..2

Figure S1: Questions from Activities of Daily Living Scale………………………………..….…………………4

Figure S2: Questions from Zarit Burden Interview Short Form (ZBI-12) ……………………………………….5

Figure S3: Questions from ET-Specific Caregiver Burden Scale…………………….…………………………..6

Figure S4. Correlations between Tremor Severity and Questionnaire Outcomes………………………...……....7

**Table S1: Demographics and Clinical Characteristics**

| **Individuals with ET (n =18)** | | **Caregivers (n = 18)** | |
| --- | --- | --- | --- |
| **Age in years, mean (SD)** | 71 (8) | **Age in years, mean (SD)** | 66 (13) |
| **Female sex, n (%)** | 7 (39%) | **Female sex, n (%)** | 12 (67%) |
| **Ethnicity, n (%)** | White: 16 (89%)  White and Indigenous: 2 (11%) | **Ethnicity, n (%)** | White: 18 (100%) |
| **Tremor duration in years, mean (SD)** | 25.7 (16.5) | **Relationship to patient, n (%)** | Spouse: 14 (78%)  Child: 3 (17%)  Partner (living apart): 1 (5%) |
| **Dominant hand treated, n (%)** | 16 (89%) | **Time between MRgFUS and follow-up caregiver questionnaires in months, mean (SD)** | 4.2 (1.4) |
| **Baseline CRST tremor score^a^, mean (SD)** | 17.8 (4.5) | **Time between follow-up CRST and caregiver questionnaires in months, mean (SD)** | 1.7 (5.3) |
| **Follow-up CRST tremor score^a^, mean (SD)** | 8.7 (6.5) |  |  |
| **Percent change in CRST tremor score, mean (SD)** | 54% (31%) |  |  |
| **Baseline CRST disability score^b^, mean (SD)** | 17.3 (4.9)^*^ |  |  |
| **Follow-up CRST disability score^b^, mean (SD)** | 7.3 (5.7)^*^ |  |  |
| **Time between MRgFUS and follow-up CRST in months, mean (SD)** | 5.9 (5.4) |  |  |
| **Co-occurring conditions** | Stroke: n = 1  Parkinson’s disease: n = 1  Normal pressure hydrocephalus: n = 1 |  |  |
| **Side effects**  **(n=12 patients in total)** | Balance/Gait Issues: n = 7 (Persisted at follow-up: n = 5)  Sensory: n = 7  (Persisted at follow-up: n = 4)  Dysarthria: n = 4  (Persisted at follow-up: n = 4)  Dysgeusia: n = 3  (Persisted at follow-up: n = 2)  Dysphagia: n = 1  (Persisted at follow-up: n = 0) |  |  |

ET, essential tremor; CRST, Clinical Rating Scale for Tremor; MRgFUS, magnetic resonance-guided focused ultrasound

**^a^** Tremor score of the treated hand was derived from the rest, posture and action/intention tasks in Part A of the Clinical Rating Scale for Tremor (CRST), along with the drawing and pouring tasks in Part B. The handwriting item was excluded to avoid skewing of data in cases where the dominant writing hand was not treated. Total possible scores for these tasks range from 0 to 28, with higher scores indicating more severe tremor.

**^b^** The disability score was derived from Part C of the CRST, which ranges from 0 to 32, with higher scores indicating more severe disability.

^*^ Data for 15 of the 18 patients were available.

**Do you currently provide your partner assistance with these activities? (yes/no)**

1. Writing (e.g., signing name)
2. Cooking (e.g., chopping veggies, setting table)
3. Walking (e.g., losing balance)
4. Transportation (e.g., operating vehicle)
5. Eating (e.g., cutting food)
6. House/yard work (e.g., vacuuming, dishes, gardening)
7. Using a computer/phone (e.g., pressing correct buttons)
8. Drinking (e.g., pouring water, drinking from cup)
9. Dressing (e.g., buttons)
10. Administering medication (e.g., setting out pills)

**Figure S1: Questions from Activities of Daily Living Scale**^2^

**Do you feel…?**

Q1: That because of the time you spend with your relative that you don’t have enough time for yourself?

Q2: Stressed between caring for your relative and trying to meet other responsibilities (work/family)?

Q3: Angry when you are around your relative?

Q4: That your relative currently affects your relationship with family members or friends in a negative way?

Q5: Strained when you are around your relative?

Q6: That your health has suffered because of your involvement with your relative?

Q7: That you don’t have as much privacy as you would like because of your relative?

Q8: That your social life has suffered because you are caring for your relative?

Q9: That you have lost your life since your relative’s illness?***

Q10: Uncertain about what to do about your relative?

Q11: You should be doing more for your relative?

Q12: You could do a better job in caring for your relative?

**Response options:** **“Never” (0), “Rarely” (1), “Sometimes (2), “Quite frequently” (3), “Nearly always” (4)**

**Figure S2: Questions from Zarit Burden Interview Short Form (ZBI-12)**

** Item was slightly modified due to technical error*

**Do you feel…?**

Q1: Worried about the assumptions others might make about your relative due to their tremor?

Q2: Embarrassed that you need to assist your relative with daily activities?

Q3: Concerned about how your relative’s illness will progress over time?

Q4: Concerned that you assist your relative more than they actually need?

Q5: Concerned that your assistance prevents your relative from achieving his/her maximum potential independence?

**Response options:** **“Never” (0), “Rarely” (1), “Sometimes (2), “Quite frequently” (3), “Nearly always” (4)**

**Figure S3: Questions from ET-Specific Caregiver Burden Scale**^2^

**Figure S4.** **Baseline, follow-up, and change correlations between tremor severity and questionnaire outcomes.** Panel A represents associations between tremor severity and assistance with activities of daily living (ADLs). Panel B represents associations between tremor severity and caregiver burden as measured by the Zarit Burden Index (ZBI)-12. Panel C represents associations between tremor severity and essential tremor-specific caregiver burden.

* *p ≤* 0.05, ***p* *≤* 0.01.
